# Investigation of an outbreak of typhoid fever in a rural district of East Malaysia, 2019

**DOI:** 10.5365/wpsar.2024.16.4.1200

**Published:** 2025-11-24

**Authors:** Jun Fai Yap, Ester Barnad, Muhammad Jikal

**Affiliations:** a Institute for Public Health, National Institutes of Health, Ministry of Health, Selangor, Malaysia.; b Sabah State Health Department, Ministry of Health, Kota Kinabalu, Sabah, Malaysia.

## Abstract

**Objective:**

Following the notification of two cases of typhoid fever to the Kudat District Health Office in February 2019, an investigation was conducted in a rural district in Sabah, East Malaysia, to determine the extent, characteristics and source of the outbreak.

**Methods:**

This epidemiological study used both active and passive case detection. Environmental samples were taken from water sources, food-handling areas and waste sites, and were analysed for the presence of *Salmonella enterica* serotype Typhi as part of the standard protocol during an announced typhoid fever outbreak. Clinical specimens underwent culture and sensitivity testing, with samples that were positive for *S.* Typhi analysed using pulsed-field gel electrophoresis to determine clonal relationships.

**Results:**

A total of 35 cases of typhoid fever were identified during 3 months. Twenty-eight cases (80.0%) occurred among Sabah’s indigenous ethnic groups, and 12 of these (34.3%) occurred in people aged 7–17 years. The index case, along with six other cases, had a history of consuming smashed fried chicken from a local restaurant. Analysis revealed three clonal clusters of *S.* Typhi isolates, with a dominant pattern found in 18 cases, which included the index case and a food handler from the implicated restaurant. Two paediatric patients experienced a relapse after initial treatment with intravenous antibiotics.

**Discussion:**

The source of the outbreak was most likely the infected restaurant worker who prepared chicken without wearing gloves; transmission most likely occurred through contaminated food or surfaces. Immediate steps to control the outbreak included chlorinating water wells, disinfecting waste disposal areas and promptly vaccinating all food handlers, including those working in street food settings. Recommendations for preventing future outbreaks include strengthening surveillance systems for acute gastroenteritis, conducting education campaigns to promote safe food-handling practices and implementing measures to improve vaccination coverage against typhoid fever among food handlers.

Typhoid fever is a bacterial infection primarily caused by *Salmonella enterica* serotype Typhi (*S.* Typhi). The incubation period is 1–2 weeks on average, following which gastrointestinal symptoms may develop, comprising abdominal distension, diffuse abdominal pain, and constipation and/or diarrhoea. Other systemic symptoms include prolonged fever, general malaise, skin rash (i.e. rose spots), myalgia and headache. (*1*) If left untreated, severe organ-specific complications may ensue, including delirium, stupor, myocarditis, intestinal haemorrhage, bowel perforation or overwhelming sepsis leading to death. (*2*)

A proportion of typhoid fever survivors become asymptomatic, chronic carriers of *S.* Typhi. The presence of asymptomatic carriers presents challenges for controlling this disease. Carriers can shed the bacteria in their stool for many years, often harbouring the *S.* Typhi bacterium on cholesterol gallstones in their gallbladder, where they form biofilms. (*3*) Bacteria excreted by carriers can exhibit multiple genetic variations, which further complicates source-tracing and the control of outbreaks. (*4*)

Humans are the sole reservoir of *S.* Typhi, and transmission occurs primarily through contaminated food or water. Although antimicrobial treatments have significantly reduced the incidence of typhoid fever in high-income countries, it remains endemic in lower-income countries, including in Fiji, Malaysia and the Philippines in the World Health Organization’s Western Pacific Region. (*5*-*7*) In these countries, ongoing challenges with sanitation, health-care access and public health infrastructure contribute to the endemicity of this communicable disease.

In Malaysia, typhoid fever is a notifiable disease under the Prevention and Control of Infectious Diseases Act 1988 and must be reported to the nearest district health office within 7 days of a diagnosis. The annual incidence typically lies in the range of 10.2–17.9 cases per 100 000 population; (*8*) however, higher rates have consistently been recorded in East Malaysia, particularly in Sabah, a state located on the island of Borneo, where the disease remains endemic. Although in Malaysia typhoid fever is predominantly a rural disease associated with reduced access to clean water and sanitation, outbreaks have occurred in rapidly urbanizing areas, where cases have been linked to street food vendors and restaurants with poor hygiene. (*8*)

On 17 February 2019, the Kudat District Health Office, in a largely rural district of Sabah state, received notification of a laboratory-confirmed case of typhoid fever. When a food handler also tested positive for *S.* Typhi at the district hospital on 21 February 2019, the national criteria for declaring an outbreak were met. This led to the declaration of the first outbreak of typhoid fever in Kudat on 22 February 2019.

The aim of this report was to describe the outbreak investigation, the sociodemographic characteristics of the cases and the control measures that were implemented once the source of the outbreak had been identified. Based on these findings, the report also recommends actions to mitigate future outbreaks.

## Methods

### Study design

This is a descriptive epidemiological study of individuals with confirmed *S.* Typhi infections.

### Epidemiological investigation

A case was defined as any individual presenting with fever or constitutional symptoms in the Kudat district of Sabah state between 20 January and 27 May 2019 (when the outbreak was declared over) and whose clinical specimen tested positive for *S.* Typhi. A carrier was defined as an asymptomatic contact whose clinical specimen tested positive for the bacterium. (*9*)

Both active and passive case-finding strategies were employed. Active case detection involved interviewing all identified cases and their close contacts using a standardized form for food- and waterborne diseases. Close contacts, regardless of whether they were symptomatic, provided samples for stool culture and testing for *S.* Typhi. Passive case detection was conducted by alerting health facilities in the district to report any suspected cases of typhoid fever. Furthermore, all health clinics and nearby hospitals were issued directives to collect stool samples from all cases of acute gastroenteritis presenting with fever. These cases were notified to the Kudat District Health Office and referred for early disease control measures. Patients experiencing severe diarrhoea were also referred for treatment and hospital admission.

Questionnaires were used to collect data about the date of symptom onset, sociodemographic characteristics (e.g. sex, age, ethnicity, occupation, citizenship) and potential sources of exposure. Cases were also asked about their food consumption during the 2 weeks before symptom onset, particularly whether they ate at restaurants.

### Environmental investigation

Combined inspections, comprising licensing visits (to verify restaurant licence status, as irregular renewals may compromise cleanliness standards), environmental sampling and food standards assessments, were carried out in various locations within the district. Water and food samples were collected from water sources, food-handling areas of restaurants and waste disposal sites and tested for the presence of *S.* Typhi at the Public Health Laboratory in the Kota Kinabalu district of Sabah state. All restaurants with an identified epidemiological link to the confirmed cases were included in the environmental investigation. This targeted approach ensured comprehensive assessment of all potential sources of foodborne infection and helped to trace possible routes of transmission within the affected areas.

### Laboratory investigation

Culture and sensitivity tests for *S. enterica* Typhi and other *Salmonella* species were conducted on clinical specimens (i.e. blood or stool samples) from suspected cases and close contacts of confirmed cases identified through active case detection, as well as from symptomatic individuals presenting at health facilities. Isolation of  *S.* Typhi from a clinical sample was considered a positive laboratory result. Positive samples were then subjected to pulsed-field gel electrophoresis (PFGE) to identify clonal relationships between the isolates.

## Results

**Fig. 1:**
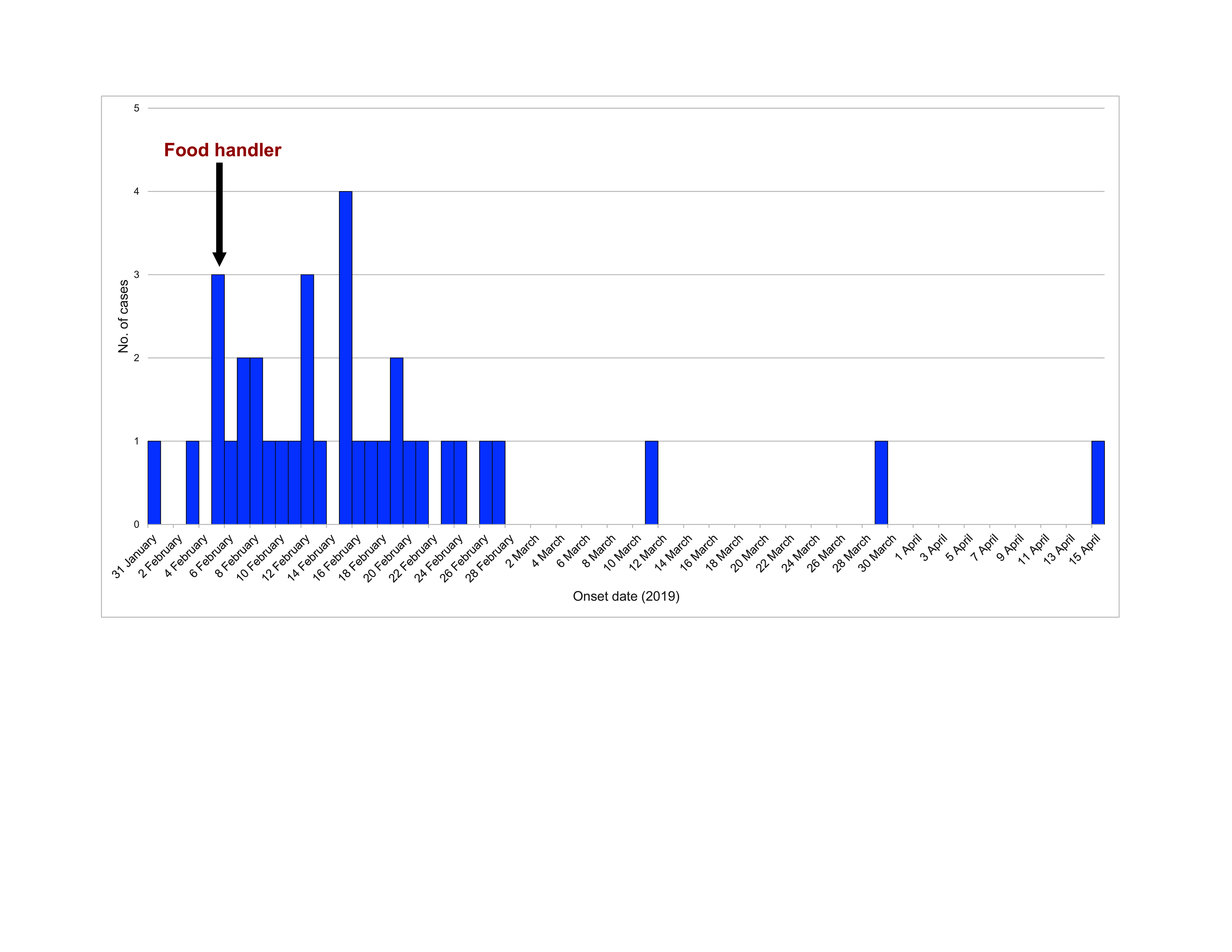
Epidemic curve of an outbreak of typhoid fever, Kudat district, Sabah, East Malaysia, February-April 2019 (*N* = 35)

A total of 35 cases of typhoid were notified in Kudat district. Symptom onset of the index case was on  31 January 2019; the last date of symptom onset was 15 April 2019 (**Fig. 1**). The index case was a 3-year-old boy who was seen at Kudat Hospital on 13 February 2019 with fever, diarrhoea, abdominal pain, vomiting and loss of appetite. A blood culture sample subsequently tested positive for *S.* Typhi, and the result was reported to the Kudat District Health Office on 17 February 2019.

[**insert** Figure 1]

During the course of the 3-month outbreak, a total of 67 cases of acute gastroenteritis were reported to the Kudat District Health Office, including the index case. Clinical samples were obtained from all 67 cases, of which 35 (52.2%) were confirmed as positive for *S.* Typhi. All cases but one (97.1%) were Malaysian citizens, and 28 (80.0%) were of Sabah native ethnicity (**Table 1**). Around half of the confirmed cases were male (18, 51.4%). Children aged 0–17 years accounted for a large proportion of the cases (17, 48.6%). All  35 cases presented with fever and were hospitalized, but none died. Other common symptoms included diarrhoea  (17, 48.6%), abdominal pain (16, 45.7%), dizziness  (15, 42.9%) and malaise (15, 42.9%).

**Table 1:** Characteristics of 35 laboratory-confirmed cases of *Salmonella enterica* serotype Typhi infection, Kudat district, Sabah, East Malaysia, February–April 2019

Characteristic (no. of respondents)	No.	%
**Sex (*n* = 35)**		
**Male**	**18**	**51.4**
**Female**	**17**	**48.6**
**Age group (years) (*n* = 35)**		
**0–6**	**5**	**14.3**
**7–12**	**5**	**14.3**
**13–17**	**7**	**20.0**
**18–55**	**16**	**45.7**
** > 55**	**2**	**5.7**
**Ethnicity (*n* = 35)**		
**Sabah native**	**28**	**80.0**
**Chinese**	**3**	**8.6**
**Iban**	**2**	**5.7**
**Malay**	**1**	**2.9**
**Jawa Banjar**	**1**	**2.9**
**Occupation (*n* = 30)**		
**Student**	**13**	**43.3**
**Employed**	**14**	**46.7**
**Unemployed**	**3**	**10.0**
**Citizenship (*n* = 35)**		
**Malaysian**	**34**	**97.1**
**Non-Malaysian**	**1**	**2.9**
**Symptoms (*n* = 35)**		
**Fever**		
**Yes**	**35**	**100.0**
**No**	**0**	**0**
**Diarrhoea**		
**Yes**	**17**	**48.6**
**No**	**18**	**51.4**
**Abdominal pain**		
**Yes**	**16**	**45.7**
**No**	**19**	**54.3**
**Dizziness**		
**Yes**	**15**	**42.9**
**No**	**20**	**57.1**
**Malaise**		
**Yes**	**15**	**42.9**
**No**	**20**	**57.1**
**Loss of appetite**		
**Yes**	**14**	**40.0**
**No**	**21**	**60.0**
**Nausea or vomiting**		
**Yes**	**13**	**37.1**
**No**	**22**	**62.9**
**Headache**		
**Yes**	**11**	**31.4**
**No**	**24**	**68.6**

All 35 cases were identified through passive case detection. Cases were found in 20 villages in this rural district. Notably, the index case and six other cases had a history of consuming smashed fried chicken (or *ayam penyet*) at a local restaurant, where a food handler worked who tested positive on 21 February 2019, suggesting a common source of enteric infection.

A total of 202 environmental swab samples and 98 food samples were collected, mainly from restaurants, but all were negative for the bacterium. One food sample, identified as mud clams (or *lokan*), tested positive for *S.* Oslo. Of the 113 water samples tested, none were positive for *S.* Typhi. However, environmental inspections revealed poor sanitation practices and inadequate handwashing facilities in restaurants, including the one where the food handler who tested positive for *S.* Typhi worked.

Altogether, three (0.5%) of the 584 stool samples collected from close contacts of the 35 confirmed cases were positive for *S.* Typhi (data not shown). Eleven (1.9%) samples were positive for other *Salmonella* serotypes, including *S.* Weltevreden (*n* = 8), *S.* Lexington (*n* = 2) and *S.* Ohio (*n* = 1) (data not shown). Despite being asymptomatic, the three contacts who tested positive for *S.* Typhi were treated with a full course of intravenous antibiotics in hospital.

**Fig. 2:**
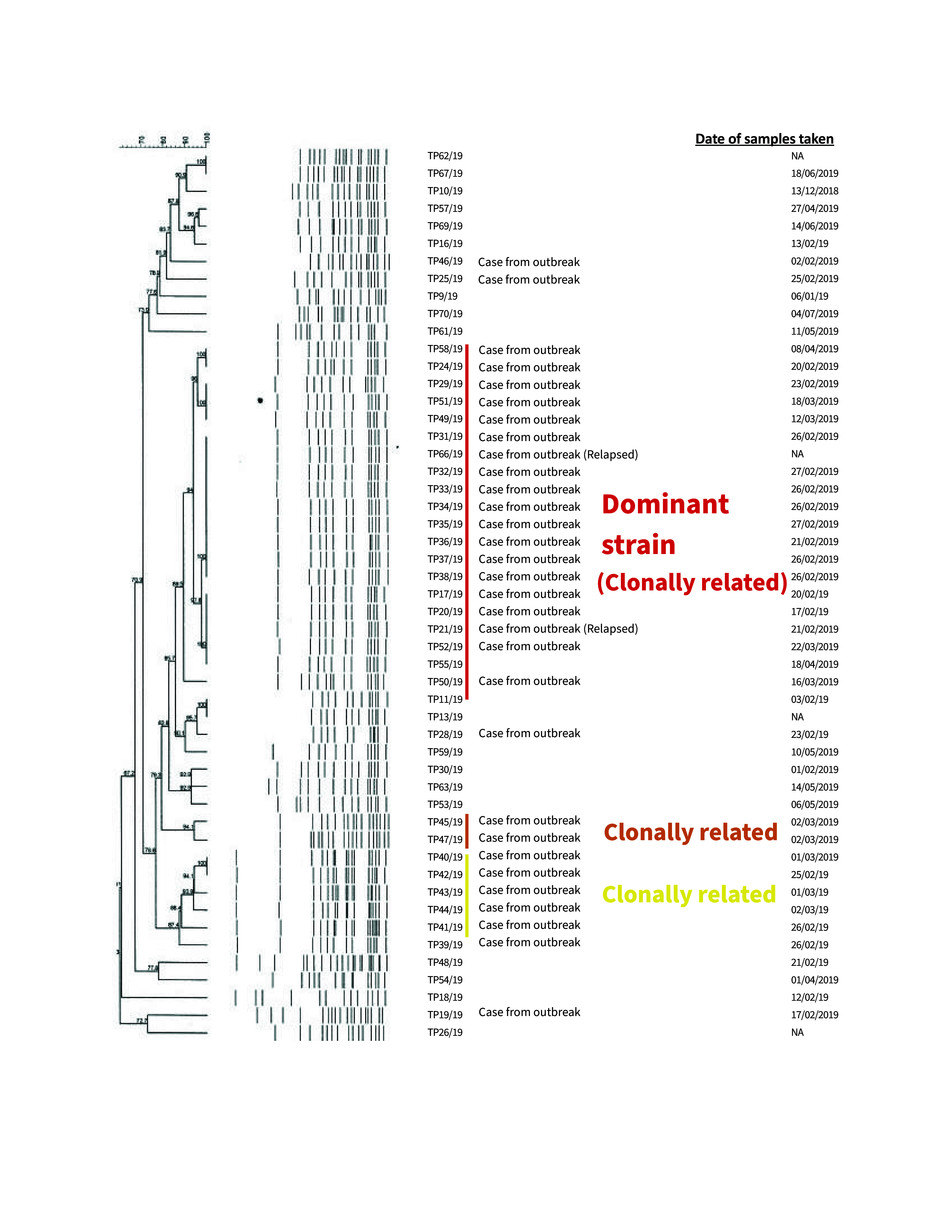
Dendogram of pulsed-field gel electrophoresis results for *Salmonella enterica* serotype Typhi, representing 31 samples from 30 cases, Kudat district, Sabah, East Malaysia, 2019

The PFGE dendrogram was based on 31 samples from 30 cases; it identified three clonal clusters of *S.* Typhi isolates, with a dominant pattern found in  19 samples (**Fig. 2**). Notably, the PFGE analysis showed that the food handler and 17 other cases, including the index case and one relapsed case, shared the same dominant clonal pattern, confirming community-wide transmission. The other two clonal patterns were observed in two and five cases. The presence of three distinct PFGE patterns suggests that the outbreak may have resulted either from multiple sources or from the introduction of three different strains at different times. However, the predominance of a single PFGE pattern indicates a primary source of infection, likely contributing to the sustained transmission within the community, and it strengthens the hypothesis implicating the restaurant where the food handler worked as a likely source of the outbreak.

[**insert** Figure 2]

Having pinpointed restaurants as potential sources of typhoid fever, control measures were promptly implemented in all affected localities. These included chlorinating domestic water wells, disinfecting public toilets and sanitizing waste disposal areas. These measures were supported by health education campaigns that highlighted the importance of safe food-handling practices, directed at both restaurant workers and the general public. Steps were also taken to strengthen surveillance for and reporting of cases of acute gastroenteritis in local health facilities to improve early detection of suspected cases of typhoid fever.

Post-investigation follow-up actions included implementing regular inspections of food establishments to ensure compliance with advice regarding sanitation and hygiene practices. A formal re-evaluation of the food establishments, conducted by public health officers, revealed improvements in cleanliness. Typhoid vaccinations were strictly mandated for all food handlers, including street food vendors. Education programmes continued, focusing on raising community awareness about typhoid fever and emphasizing the importance of consuming safe food and water. After approximately 3 months, on 27 May 2019, the typhoid outbreak was officially declared over.

Two paediatric patients relapsed after their initial antibiotic treatment and hospital discharge. The first relapsed case was the index case, who was initially treated with intravenous ampicillin (25 mg/kg four times per day for at least 1 week), but was readmitted within 3 months with abdominal pain; a repeat blood culture confirmed *S.* Typhi infection, and he was treated with intravenous ceftriaxone (100 mg/kg twice per day for 2 weeks). The second relapsed case was a 13-year-old boy originally treated with intravenous ceftriaxone (2 g/day for 2 weeks). He too was readmitted within 3 months with fever, diarrhoea and abdominal pain. The presence of *S.* Typhi was confirmed via repeat blood culture, and he was treated with intravenous meropenem (1 g three times per day for 1 week) before being discharged for a second time. Unfortunately, due to limited resources, blood samples were not analysed further to compare the first and second infections.

## Discussion

Our investigation suggested a common-source outbreak, most likely epidemiologically linked to an infected food handler working at a restaurant where the index case and a further six cases reported consuming smashed fried chicken. However, the sources of the additional 27 cases remain unclear. The food handler performed kitchen tasks, such as preparing and cutting chicken, but did not wear gloves while performing these tasks. This raises the possibility that the transmission of *S.* Typhi bacteria could have occurred through direct contact with contaminated food items or surfaces. The lack of proper handwashing facilities in the restaurant may have facilitated the spread of the pathogen.

This study observed a high prevalence of cases in school-aged children, a trend that aligns with findings from other countries. (*10*) A high prevalence of typhoid fever in children has been attributed to the fact that good hygiene habits, such as handwashing, are often less well developed in children. In this setting, malnutrition, which weakens the immune system and is common among children aged 5–12 years in Sabah, was likely an additional contributory factor. (*11*)

Practising good personal hygiene and regular handwashing and maintaining proper sanitation reduces the transmission of *S.* Typhi among vulnerable populations. Other important control measures include ensuring strict adherence to contact protocols in clinical settings; such precautions should remain in place throughout a patient’s hospitalization, until three consecutive stool cultures, taken 48 hours after completing antibiotic therapy, are confirmed as negative. (*9*)

Vaccination against typhoid, which can prevent at least 50% of cases of typhoid fever during the first 2 years after vaccination, also has a role to play, (*12*) and in Malaysia it is mandatory for all food handlers. Regulations for food handlers also stipulate that they should have booster doses every 3 years, as immunity is not lifelong. However, the lack of surveillance data and lack of enforcement of vaccination record-keeping means that it is difficult to assess compliance with regulations, but it is likely that coverage of typhoid vaccine is suboptimal among food handlers in restaurant settings. Moreover, vaccination is not 100% effective and ideally should be complemented by other nonpharmacological measures to prevent transmission. (*13*)

The relapses in two paediatric patients after their initial antibiotic treatment could have been due to either re-exposure to contaminated sources after their discharge from hospital or an inadequate immune response. Additionally, if the patients had developed a chronic carrier state, that may have allowed bacteria to persist and cause a recurring infection. Determining the precise cause of these relapses would require further genotypic investigation.

Since the outbreak, laboratory capacity has been enhanced to support more detailed molecular epidemiology studies, including introducing whole genome sequencing, which will facilitate better understanding of the genetic diversity of *S.* Typhi in Sabah in the future.

That cases relapsed, despite adherence to clinical protocols during initial admission, including the requirement for three negative stool samples before discharge, raises concerns about the possibility of multidrug-resistant *Salmonella*. The bacterium is considered multidrug-resistant if it is resistant to at least three broad-spectrum antibiotics used empirically for treatment, such as ampicillin, chloramphenicol and co-trimoxazole. (*14*) Extensively drug-resistant *Salmonella*, however, has evolved to additionally resist fluoroquinolones or even third-generation cephalosporins. This growing antibiotic resistance highlights a critical challenge in treating typhoid fever. Malaysia has yet to report any instances of extremely drug-resistant *Salmonella*; (*15*) nonetheless, there is the potential for additional undetected cases or importations of resistant bacteria.

The outbreak investigation faced several limitations, including the inability to conduct whole genome sequencing to compare the Sabah *S.* Typhi isolates with those from other areas, particularly neighbouring countries, such as Indonesia and the Philippines. Additionally, due to resource constraints, not all samples were subjected to PFGE. This constraint, in particular, limited understanding of the outbreak's molecular epidemiology and the ability to verify the source of the outbreak. Underreporting of cases through passive surveillance and potential recall bias during active case detection also posed significant limitations. In addition, the use of a standardized questionnaire prevented the collection of data about other risk factors, such as handwashing habits and food preparation practices. These limitations, combined with other logistical constraints, such as challenges in identifying an appropriate control group, precluded the use of a more robust approach to establish causality.

### Conclusions

This outbreak investigation identified 35 laboratory-confirmed cases of typhoid fever, a high proportion of which occurred in children. All cases presented with fever and required hospitalization, but there were no fatalities. Results from the PFGE analyses linked the outbreak to a food handler at a local restaurant, indicating common-source transmission. The swift implementation of control measures effectively curtailed further spread. Nevertheless, it is crucial to ensure ongoing vigilance and to enhance existing public health infrastructure. This outbreak highlights the urgent need for vaccination against typhoid among food handlers.
